# Public Awareness and Perceptions of Antibiotic Use in Human and Veterinary Medicine in Serbia

**DOI:** 10.3390/antibiotics14050523

**Published:** 2025-05-19

**Authors:** Gordana Belamarić, Dejana Vuković, Zoran Bukumirić, Rada Sandić Spaho, Gordana Marković

**Affiliations:** 1 Institute of Public Health of Belgrade, 11000 Belgrade, Serbia; belamaric.gordana@gmail.com; 2 Institute of Social Medicine, Faculty of Medicine, University of Belgrade, 11000 Belgrade, Serbia; dejana.vukovic@med.bg.ac.rs; 3 Institute for Medical Statistics and Informatics, Faculty of Medicine, University of Belgrade, 11000 Belgrade, Serbia; zoran.bukumiric@med.bg.ac.rs; 4 Faculty of Nursing and Health Science, Nord University, 8049 Bodø, Norway; 5 Faculty ECM, Alma Mater Europaea University, 2000 Maribor, Slovenia; gm5rovic@gmail.com

**Keywords:** antibiotics, antimicrobial resistance, One Health, excessive use of antibiotics

## Abstract

**Background/Objectives**: Antimicrobial resistance (AMR) is a growing global health threat, requiring an approach that integrates human, animal, and environmental health. Public awareness and responsible antibiotic use are key to combating AMR. This study examines the knowledge, attitudes, and practices related to antibiotic use among the general population in Serbia, exploring their impact on antimicrobial stewardship efforts. **Methods**: A cross-sectional study was conducted in December 2022 using a three-stage stratified random sample of the Serbian population. The Eurobarometer questionnaire was utilized with permission from the European Commission. Statistical analyses included multivariate ordinal logistic regression to identify predictors of desirable attitudes and behaviors related to antibiotic use. **Results**: Almost two-thirds of respondents (61.6%) reported not receiving any advice on rational antibiotic use, underscoring the need for education of the general population and raising awareness. A Higher Antibiotic Knowledge Score was associated with a 60% greater likelihood of favorable attitudes and a 21% greater likelihood of rational antibiotic use. Pharmacists were identified as the primary source of antibiotic-related information for the general population in Serbia, but there is limited public interest and awareness of antibiotic use in animals, as well as of the bans on using antibiotics to stimulate growth in farm animals within the EU. **Conclusions**: Through targeted educational interventions, strengthening interdisciplinary collaboration and AMR control measures is necessary for human and veterinary medicine. Although pharmacists are the primary source of information about antibiotics for the population in Serbia, collaboration with physicians and their empowerment should be introduced to strengthen antimicrobial stewardship further.

## 1. Introduction

Antimicrobial resistance (AMR), one of the leading health challenges of the 21st century, should be approached considering the interdependence between the human, animal, and environmental health dimensions [1]. There is a growing concern among researchers and stakeholders that the environment acts as an AMR reservoir and could potentially have a key role in disseminating antimicrobial resistance [2]. In addition to the importance of the rational use of antibiotics in the human population, the problem of excessive antibiotics usage in domestic animals, especially those further used in human nutrition, is considered one of the most significant factors in the spread of AMR [3]. A “One Health” approach aims to achieve optimal health for people, domestic animals, wildlife, plants, and the environment, making comprehensive knowledge and counseling about antibiotic use the essential tools for addressing antimicrobial resistance effectively [4]. Activities in the field of AMR that are undertaken mainly in individual sectors are not sufficiently effective, but implementing the “One Health” approach principles, combining cross-sectoral and interdisciplinary collaboration, could improve health outcomes for people, animals, plants, and the shared environment [1,5].

Researching knowledge, attitudes, and practices concerning antibiotic use in the three-staged representative sample of the general population of the Republic of Serbia, this study analyzes the possible impact of knowledge, both on the attitude and on antibiotic utilization. Bearing in mind different research and reviews about antibiotic use in human and veterinary medicine [6,7,8], the main goal is to highlight the understanding of the implications of the inappropriate use of antibiotics across these two sectors and empower people to become active participants in strengthening public health efforts and combating antimicrobial resistance through informed decision-making and responsible practices. Serbia has universal healthcare coverage through the public system and the National Health Insurance Fund. Still, access and quality may vary, and therefore, sometimes people combine public coverage with out-of-pocket payments in healthcare [9,10,11]. The network of 12 accredited veterinary institutes in Serbia is responsible for epidemiological surveillance and laboratory testing of the safety of animal-origin food and animal feed [12]. Although there are positive aspects of prescribing practices, data also reveal that Serbia is among the European countries with the highest antibiotic consumption [12].

Improving knowledge and antimicrobial stewardship in human and animal health may help to reduce AMR, but raising public awareness and education is critical for preserving the effectiveness of existing antimicrobials [13]. To the best of our knowledge, this is the first study in the Republic of Serbia to explore public attitudes and awareness regarding the use of antibiotics in animals. However, a survey conducted previously among veterinarians in Serbia showed that, while they generally have a positive attitude toward the selective use of antimicrobial drugs in animals, less than a third of the surveyed veterinary doctors had even heard of the term “antimicrobial stewardship” [14].

## 2. Results

A total of 1014 respondents participated in the study, with a balanced regional distribution across Serbia and a slight majority of women (54.2%). Most respondents reported moderate financial status and good self-rated health. Participants’ employment status was diverse, with nearly half employed in the private sector (48.7%), followed by those in the public sector (23.8%). Unemployed individuals accounted for 13.2%, while pensioners comprised 10.1% of the sample. Students and farmers were the least represented groups, comprising 3.0% and 1.3%, respectively.

### 2.1. Attitudes Towards the Use of Antibiotics

#### 2.1.1. Predictors of the Desirable Attitude Towards Antibiotics Usage

Almost two-thirds of all respondents, 625 (61.6%), did not receive any advice on the rational use of antibiotics in the last 12 months. The remaining, about a third of the participants in the research that received the advice about antibiotics, further declared whether this advice has changed their opinion related to antibiotic use. Respondents who received advice and stated that this advice changed their opinion on antibiotic use were further analyzed to determine whether they had a desirable or undesirable attitude toward antibiotic use. Based on the information and advice about the use of antibiotics, the respondents reflected on how they will use antibiotics in the future. Desirable attitude towards antibiotic use was expressed with the following four answers: I will always consult a doctor when I think I need antibiotics; I will no longer self-medicate with antibiotics; I will no longer take antibiotics without a prescription from a doctor; I will no longer keep leftover antibiotics for next time I am ill. In addition, the following two answers reflected an undesirable attitude: I will give leftover antibiotics to my relatives or friends when they are sick; Don’t know. Respondents’ knowledge about antibiotics was assessed through answers to four questions, and an Antibiotic Knowledge Score (AKS) was rated from 1 to 4, depending on the number of correct answers [15].

**Figure 1:**
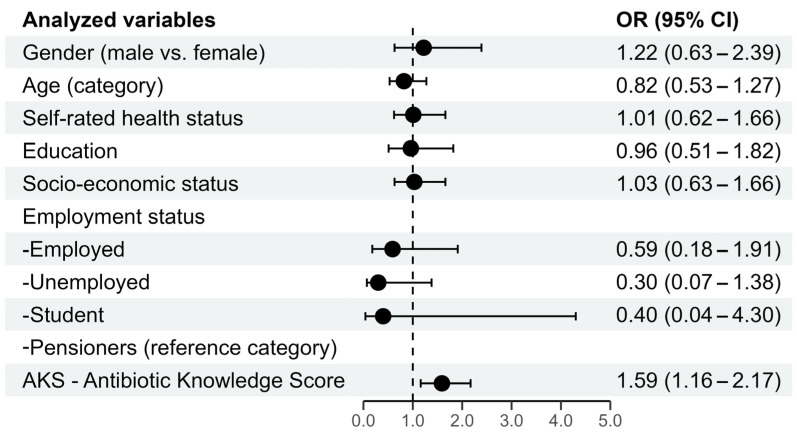
Graphics presentation of the ordinal logistic regression with the predictors of the desirable attitude towards antibiotic use.

Multivariate ordinal logistic regression was used to identify predictors that could potentially be associated with a desirable attitude toward the use of antibiotics. In the multivariate ordinal logistic regression model, a statistically significant predictor of a desirable attitude in the use of antibiotics is the Antibiotic Knowledge Score (*p* = 0.004), with OR = 1.59, which shows that respondents with higher knowledge score have almost 60% higher odds for a desirable attitude regarding antibiotic use while controlling for all other factors in the model (Figure 1).

#### 2.1.2. Attitude About the Cessation of Antibiotic Treatment

Although the vast majority of the respondents, 868 in total (85.6%), know that they should stop taking antibiotics only when they have taken all prescribed antibiotics, per the doctor’s instructions, every tenth respondent, for a total of 105 (10.4%), potentially stops taking antibiotics when feeling better (Table 1).

#### 2.1.3. Attitudes About Trustworthy Sources of Information About Antibiotics

**Figure 2:**
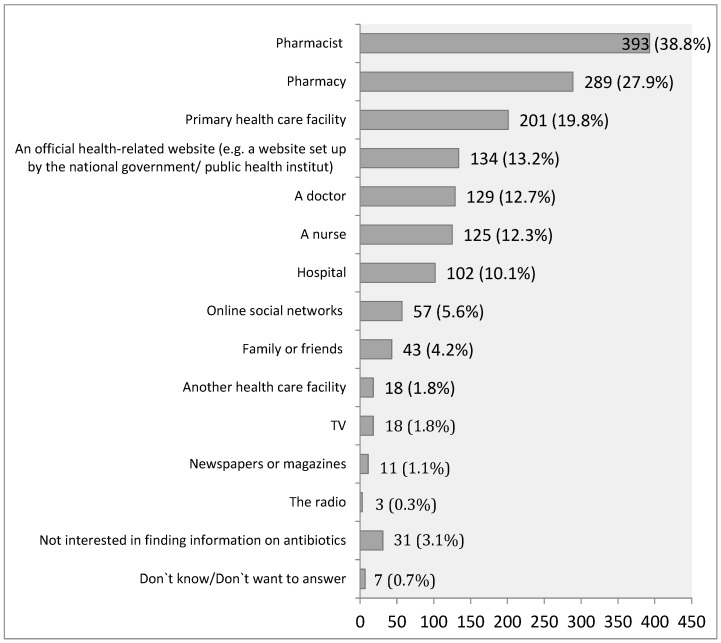
Trustworthy sources of information about antibiotics in the population of the Republic of Serbia.

As a trustworthy source of information on antibiotics, the most significant number of respondents in Serbia would use pharmacies 289 (27.9%) and pharmacists 393 (38.8%). A smaller number of them would use doctors 129 (12.7%) and nurses 125 (12.3%) as a trustworthy source of information about antibiotics (Figure 2).

#### 2.1.4. Attitudes About the Antibiotics Use in Farm Animals

Concerning attitudes of the population of the Republic of Serbia towards the use of antibiotics on sick animals, i.e., animals used for consumption (meat, dairy products, etc.) and awareness of the general population about the ban on using antibiotics to stimulate growth in farm animals, just over one-third of the respondents, a total of 382 (37.3%), know that the use of antibiotics to stimulate growth in farm animals is banned in the EU. In contrast, almost two-thirds of respondents do not know this fact, 632 (62.3%). Most respondents believe that sick animals should be treated with antibiotics if this is the appropriate treatment, but 22.4% of the research participants do not know (Table 2).

### 2.2. Predictors of the Desirable Behavior Related to Antibiotic Use

The respondents’ antibiotic usage behavior was categorized as desirable or undesirable based on how they used antibiotics. Desirable behavior was taking antibiotics with a doctor’s prescription. In contrast, undesirable behavior included taking antibiotics without a prescription, using leftover antibiotics from a previous treatment, obtaining antibiotics from a pharmacy without a prescription, or acquiring antibiotics through other non-prescribed means.

**Figure 3:**
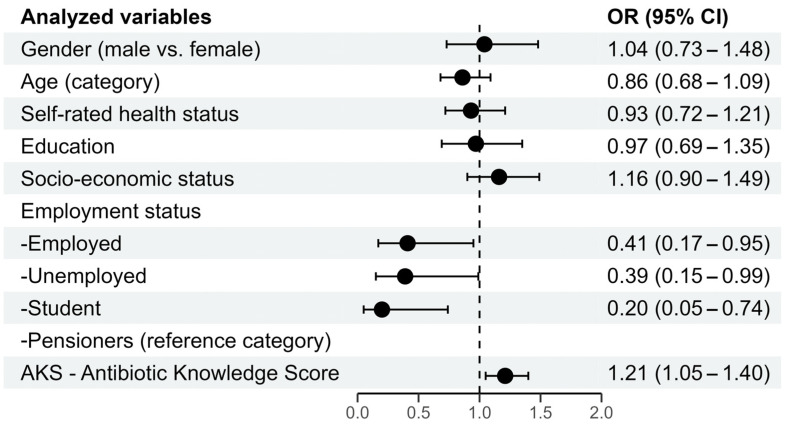
Graphic representation of the logistic regression of predictors of desirable behavior in antibiotic use.

Multivariate ordinal logistic regression was used to identify predictors that could potentially be associated with a desirable behavior related to antibiotic use. The model comprises seven predictors, which include gender, age of the respondents, health status, education level, socioeconomic status, employment status, and Antibiotic Knowledge Score (Figure 3). The statistically significant predictors of the desirable behavior regarding antibiotic use are employment status [employed (B = −0.900; *p* = 0.038), unemployed (B = −0.951; *p* = 0.047), and students (B = −1.613; *p* = 0.016) compared to pensioners as the reference category]. Employed and unemployed individuals have a nearly 60% lower chance of desirable behavior than pensioners. In comparison, students have an 80% lower chance for desirable behavior, controlling for all other factors in the model. Knowledge score (B = 0.193; *p* = 0.008), with an odds ratio OR = 1.21, indicates that respondents with each additional knowledge score have a 21% greater chance of demonstrating desirable behavior, controlling for all other factors in the model.

### 2.3. Interest in Different Topics About Antibiotic Use

**Figure 4:**
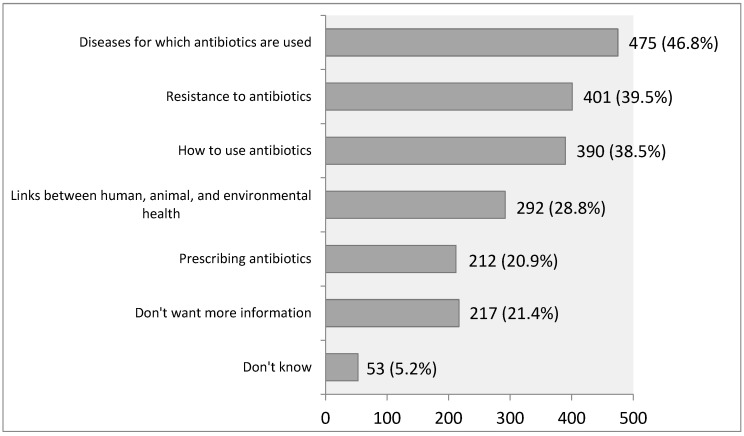
Acquiring more information on specific topics about antibiotic use.

Concerning the distribution of respondents’ interest in specific topics related to the use of antibiotics (while they had an opportunity to give multiple answers to this question), the most significant number of participants wanted more information about diseases for which antibiotics are used, for a total of 475 respondents (46.8% of the total number of respondents), than about antibiotic resistance, with a total of 401 (39.5%), and how to use antibiotics, for 390 of all respondents (38.5%) (Figure 4).

## 3. Discussion

Since almost two-thirds of the respondents from the general population in Serbia did not receive any advice on the rational use of antibiotics, it is necessary to increase the proportion of the population with access to such information and education. According to Eurobarometer data [16], the situation regarding access to advice about the rational use of antibiotics is similar in the EU, with an average of one-third of the population acquiring some advice about antibiotic use. In line with Eurobarometer findings, our research underscores the critical need for education and timely access to information to empower individuals in making informed decisions about antibiotic use, their health, and overall well-being.

Studies of knowledge, attitudes, and practices (KAP) concerning antibiotics and antimicrobial resistance are essential for improving antimicrobial management and preventing the spread of antimicrobial resistance, providing an evidence-based foundation for designing AMR control programs and raising public awareness about the rational use of antibiotics [17,18,19]. Our research shows that the working-age population has a 60% lower chance of exhibiting desirable behavior than pensioners, while students have an even 80% lower chance than pensioners. Young people and the working-age population in Serbia are priority target groups for specially designed educational interventions on the rational use of antibiotics. Respondents with better knowledge are more likely to develop desirable attitudes and behaviors toward antibiotic use. All of the above supports the need for a more personalized approach—not only in antibiotic treatment and administration, which has already been recognized by other authors [20]—but also in education about antibiotics and the use of new technologies for more advanced analyses of the impact of various determinants and characteristics on attitudes and behavior related to antibiotic use [21]. Future research should further explore the knowledge and attitudes of the general population regarding antibiotic use in animals, with a particular focus on empowering the working-age population and young people to learn more about rational antibiotic usage. Since we identified the target groups, interventions should be designed accordingly, utilizing approaches such as online support and other digital solutions specifically designed to engage young and working-age people.

A relatively recent approach to controlling AMR is using Point-of-Care Interventions (POCIs) as strategies implemented in primary healthcare [22]. These interventions could play a crucial role in bridging the gap between human and animal health as a part of the One Health approach by providing tailored training to veterinarians and medical practitioners on the responsible use of antibiotics in animal health and the prevention of antimicrobial resistance [22,23]. Incorporating medical history, lifestyle characteristics, risk factors, and other relevant aspects could facilitate and enhance a more personalized approach to planning intervention strategies for combating AMR. This trend is increasingly cited in the literature as both significant and promising for the future [24].

Although the majority of the respondents in the study know that they should stop taking antibiotics only when they have taken all prescribed antibiotics, every tenth respondent stops taking antibiotics when feeling better, which is identified as a contributing factor to the spread of antimicrobial resistance [25,26]. Other studies have also reported a similar trend, with a portion of the surveyed population exhibiting this behavior. For instance, in a study conducted among pharmacy users in China, less than one-fifth of all participants always completed their entire antibiotic course as prescribed [27]. Nevertheless, our study demonstrates that better knowledge significantly predicts a positive attitude toward antibiotic use, increasing the likelihood of a favorable attitude by 60%. Additionally, it enhances the probability of exhibiting responsible behavior by 21%.

Results of our study showed that most people in Serbia receive information about antibiotic use from pharmacies and pharmacists. At the same time, they are three times less likely to obtain such information from doctors or nurses. In contrast, residents of the EU are three times more likely to consider doctors as a reliable source of information on antibiotics compared to pharmacists [16]. One of the key findings in this and other research, which is both applicable and relevant to other countries, including Serbia, is the importance and necessity of close collaboration with physicians who prescribe antibiotics in order to achieve long-term improvements in antimicrobial stewardship [28,29,30]. This systematic long-term approach results in Sweden having some of the lowest antibiotic consumption rates and antimicrobial resistance levels in the European Union (EU) and globally [16,18].

Respondents in our research showed less interest in the links between human, animal, and environmental health related to antibiotics than in the aspects of antibiotic use in the human population. This imposes the need to bring topics such as the use of antibiotics in animals and the spread of resistant strains of bacteria through the environment closer to the general population. The minority of the respondents from the general population of the Republic of Serbia know that the use of antibiotics to stimulate growth in farm animals is banned in the EU. While most respondents think that sick animals should be treated with antibiotics if appropriate, researchers underline that those medications should always be used in animals under the strict supervision of a veterinarian. Whenever possible, vaccination should be used as the primary option instead of antibiotic treatment [31]. Clear communication about regulations, risks, and responsible antibiotic use in both human and veterinary medicine could help ensure a more informed general population that can actively contribute to antimicrobial stewardship efforts [32]. Future research should focus on deepening the understanding of knowledge, attitudes, and behaviors related to antibiotic use in animals, particularly identifying effective strategies for raising awareness and empowering young people and working-age population groups. Some studies recommend that countries in the European region prioritize allocating the necessary human and financial resources to tackle AMR and establish an efficient system for monitoring and evaluating the implementation of activities outlined in AMR action plans [33]. Individual countries are further encouraged to invest in comprehensive surveillance systems to thoroughly learn and understand antibiotic prescribing and consumption patterns, which will support the design of effective antimicrobial stewardship systems [34].

This study’s strength is a large, comprehensive sample from the general population of the Republic of Serbia. Online questionnaires allow access to respondents in remote areas, yet online data collection has limitations since some areas may lack internet access. This may suggest possible selection bias. Additionally, the educational structure of the respondents was more favorable compared to the average educational structure of the population of the Republic of Serbia, which could be expected when filling out an online questionnaire. Self-administered surveys have a potential risk of recall bias, as questions refer to events from the previous 12 months. A three-stage, stratified random sample of the general population partially mitigates these limitations.

## 4. Materials and Methods

The cross-sectional study was conducted in December 2022 on the three-stage sample population of the Republic of Serbia, utilizing the Eurobarometer questionnaire [16], with the permission of the Directorate-General for Communication Unit (Europe Direct reply no. 457661/2021, stating that the reuse of the document by third parties is permitted according to Commission Decision 2011/833/EU of 12 December 2011, under Article 6 of the Reuse Decision, with reuse subject to acknowledgment of the source). Approval was obtained from the Ethics Committee of the Institute of Public Health of Belgrade (V-2 no. 86/2/2022).

A total of 1014 respondents participated in the research (research database is available at the Supplementary Materials, evenly represented by regions of the Republic of Serbia. Demographic characteristics were aligned with data from the 2011 census conducted by the Statistical Office of the Republic of Serbia to ensure a representative sample of the Serbian population, reflecting an urban-to-rural distribution of approximately 60% to 40%. A stratified, three-stage random sampling approach was used: municipalities and cities were selected first, followed by local communities proportional to population size, and, finally, individuals were randomly chosen with equal probability. Post-stratification adjustments were made for potential non-response, using gender and age as control variables. Data collection was carried out through an online survey (Computer-aided Web Interviewing—CAWI), with participation being voluntary, anonymous, and based on self-completed responses to a 25-question instrument [15]. The required sample size was determined to be 634 respondents to ensure a statistically reliable evaluation of adequate knowledge frequency. This calculation was based on a 2% margin of error, a 95% confidence level, and an assumed prevalence of the studied phenomenon at 7.1% [15,35]. Despite limitations related to the online survey, which may introduce selection bias and recall issues, the study benefits from a large, three-stage, stratified, representative sample of the Serbian population.

### Statistical Methods

Results were presented as frequency (percent). Datasets for continuous numerical variables were described using mean and standard deviation, while attributive variables were described using frequency and percentage. The following tests were used to test the hypotheses on the difference between frequencies: the Chi-square Test, Fisher’s exact test, and McNemar’s Test. Predictors of the desirable attitude and desirable behavior regarding antibiotic use were analyzed by multivariate ordinal logistic regression. All *p*-values less than 0.05 were considered significant. Statistical data analysis was performed using IBM SPSS Statistics 22 (IBM Corporation, Armonk, NY, USA) and R-4.0.0 software (The R Foundation for Statistical Computing, Vienna, Austria).

## 5. Conclusions

With almost two-thirds of respondents reporting that they did not receive any advice on antibiotic use, this study underlines the necessity of providing a broader population with access to reliable information about antibiotics. The experiences of some countries [18,28] where systematic and long-term strategies have provided successful antimicrobial stewardship emphasize the importance of engaging all available resources, including healthcare professionals, pharmacists, and the public.

Findings from this study reinforce the significance of knowledge as a predictor of desirable attitudes and behaviors regarding antibiotic use. Respondents with higher Antibiotic Knowledge Scores had a greater probability for responsible and rational antibiotic use, which further underlines the importance of educational interventions. Although pharmacists are the primary source of information about antibiotics for the population in Serbia, establishing better collaboration with physicians and their empowerment could be of great importance for further strengthening antimicrobial stewardship. The findings of this study contribute valuable insights into the public perception of antibiotic use and underscore the need for broader education and raising awareness about the rational use of antibiotics, particularly among young people and the working-age population in Serbia.

Since the general population showed limited interest and awareness regarding the use of antibiotics in animals and their environmental impact, future actions should be concentrated on bridging the gap between human, animal, and environmental health. Messages and actions related to the responsible use of antibiotics in veterinary medicine are of great importance, as well as the promotion of alternative strategies such as vaccination. To gain a deeper insight into antibiotic use in human and veterinary medicine, as well as the role of the environment in AMR transmission, a qualitative study involving interviews and focus groups with healthcare professionals, veterinarians, farmers, and environmental experts could provide those valuable insights into perceptions, challenges, and opportunities for improving antimicrobial stewardship.

In terms of national policies, our results show that clear, targeted communication strategies should address gaps in population awareness about antibiotic use in animals and the environment, particularly focusing on young and working-age populations. The findings suggest that national policy should prioritize comprehensive, multisectoral, and age-specific public education campaigns for a better understanding of antibiotic use, integrating AMR topics into school curricula, professional training programs, and public media initiatives, ensuring that information reaches diverse demographic groups and fosters more responsible attitudes and behaviors.

## Figures and Tables

**Table 1:** Cessation of antibiotic treatment.

When Should You Stop Taking Antibiotics Once You Have Begun a Course of Treatment?	f	%
When I feel better	105	10.4
When I have taken all antibiotics as directed by my doctor	868	85.6
Other reasons	27	2.7
I don’t know	14	1.4

**Table 2:** Antibiotics usage in sick farm animals, i.e., animals used for consumption.

Use of Antibiotics in Animals	Categories	n	%
To what extent do you agree or disagree that sick farm animals, i.e., animals used for consumption (meat, dairy products, etc.), should be treated with antibiotics if this is the most appropriate treatment?	Totally agree	194	19.1
	Tend to agree	303	29.9
	Tend to disagree	114	11.2
	Totally disagree	176	17.4
	Don’t know	227	22.4
Did you know that using antibiotics to stimulate growth in farm animals is banned within the EU?	Yes	382	37.3
	No	632	62.3

## Data Availability

The original data presented in the study are openly available in Supplementary Materials.

## Supplementary Materials

The following supporting information can be downloaded at: https://www.mdpi.com/article/10.3390/antibiotics14050523/s1—Database of antibiotics.

## Abbreviations

The following abbreviations are used in this manuscript:

AMR	Antimicrobial Resistance
AKS	Antibiotic Knowledge Score
EU	European Union
KAP	Knowledge, Attitudes, and Practices Studies
POCIs	Point-of-Care Interventions
